# Maternal Obesity and its Short- and Long-Term Maternal and Infantile Effects

**DOI:** 10.4274/jcrpe.2127

**Published:** 2016-06-06

**Authors:** Levent Korkmaz, Osman Baştuğ, Selim Kurtoğlu

**Affiliations:** 1 Erciyes University Faculty of Medicine, Department of Pediatrics, Division of Neonatology, Kayseri, Turkey; 2 Erciyes University Faculty of Medicine, Department of Pediatrics, Division of Endocrinology, Kayseri, Turkey

**Keywords:** Maternal obesity, fetal, neonatal, short- and long-term effects

## Abstract

Obesity, in childhood or in adulthood, remains to be a global health problem. The worldwide prevalence of obesity has increased in the last few decades, and consequently, the women of our time suffer more gestational problems than women in the past. The prevalence of obesity is greater in older women than in younger ones and in women with low educational level than in their counterparts with a higher level of education. Maternal obesity during pregnancy may increase congenital malformations and neonatal morbidity and mortality. Maternal obesity is associated with a decreased intention to breastfeed, decreased initiation of breastfeeding, and decreased duration of breastfeeding. We discuss the current epidemiological evidence for the association of maternal obesity with congenital structural neural tube and cardiac defects, fetal macrosomia that predisposes infants to birth injuries and to problems with physiological and metabolic transition, as well as potential for long-term complications secondary to prenatal and neonatal programming effects compounded by a reduction in sustained breastfeeding.

## INTRODUCTION

In the United States and Europe, 20-40% of pregnant women are obese or their weight gain during pregnancy is excessively high. Body mass index (BMI) is the basic criterion when assessing obesity (Table 1) (1,2).

The studies conducted between 2001 and 2007 demonstrated that in the United Kingdom, 20% of pregnant women were obese and that obesity rose in parallel with age, parity, and socio-economic level (3). However, attention should be drawn to the fact that these figures vary in different parts of the world and in different ethnic groups. Obesity negatively affects both contraception and fertility. Maternal obesity is linked with higher rates of cesarean section as well as higher rates of high-risk obstetrical conditions such as diabetes and hypertension.

In recent years, the subject of epigenetic mechanisms having important effects on maternal obesity has become an issue of discussion. The genetic effects of maternal obesity on child development cannot be explained with the Mendelian model, which is based on genes per se. How this interaction occurs is a broad field of research (4). Epigenetics can be used to refer to various factors which collectively regulate gene expression. The most common of these is DNA methylation (5). In addition, in a study on the offspring of rural Gambian women who had been eating different diets during pregnancy depending on the season, differences have been found in DNA methylation of metastable epialleles in the peripheral blood during childhood (6).

Another theory as to how metabolic disease and obesity in children are linked is related to the epigenetic differentiation of the genes in ribonucleic acid and DNA transmission, without any change in the sequence of nucleotides induced through the in utero environment owing to inflammatory environment, insulin resistance, and other hormonal factors (7,8). Glucose intolerance due to histone modification encoding the increased glyconeogenesis and enzyme phosphoenolpyruvate carboxykinase-1 has been detected in newborn mice fed on a diet which is high in fat (9). Consistent with these observations, it was assumed that overfeeding in mice was a precursor to the epigenetic programming of insulin receptors. In addition, the programming of the estrogen receptor, mitogen-activated protein kinase, as well as tumor suppressors BRCA-1, P53, and caveolin-1 is affected by diet-induced changes (10).

Based on all these data, it has been established that birth weight, which can be assumed to reflect the expression of a genetic marker as well as the effect of the environment throughout intrauterine life on this genetic make-up, is positively correlated with adult bone mass.

For this reason, in the subsequent stages of life, maternal obesity may be associated with the risk of osteoporosis. The induction epidemic changes for this disease throughout puberty and pregnancy may play significant roles in determining the risk of cancer in subsequent stages of life (11,12).

Maternal obesity is a problem more frequently encountered in pregnancies of women of advanced age. It also leads to other problems such as gestational diabetes mellitus (GDM), pregnancy-related hypertension, preeclampsia, and sepsis (13,14). Maternal obesity is, in all probability, also associated with pre-gestational diabetes. It has been reported that the weight gain in the 5-year period prior to becoming pregnant increases the risk of GDM development, notably in women who are not obese at the outset (15). Obesity with insulin resistance, may contribute to hyperglycemia, hyperinsulinism, GDM, and other untoward perinatal consequences. However, maternal obesity is correlated with the side effects of pregnancy independent of GDM.

In obese pregnant women, the rates of births with intervention or by cesarean section, as well as the risk of intrapartum and postpartum complications are also higher (16). The levels of blood lipids, notably of triglycerides, peak at gestational weeks 31-36 in response to such gestational hormones as progesterone, 17 βE2 and placental lactogenic hormone, which also increase during pregnancy (17,18). In obese pregnant women, the increase in triglyceride levels accompanied by a temporary decrease in high-density lipoprotein is particularly interesting (19). The trans-placental transport of lipids is not yet understood. However, normal placental transport and the synthesis of lipids in women with maternal obesity and gestational hyperlipidemia can give rise to disorders that may affect fetal development and growth (20).

Insulin resistance increases progressively throughout pregnancy as a result of the continuous production of counter-regulatory hormones by the placenta. However, obese women have higher insulin resistance (lower insulin sensitivity) than women of normal weight, which results in elevated availability of lipids for fetal growth and development. In fact, there is a higher expression of genes related to lipid metabolism and transport in the placenta of obese women with GDM, which results in a higher birth weight and fat mass in their offspring. More than 50% of women with GDM become diabetic within the first 20 years after giving birth (21,22). As for amino acids, they do not rise in the first trimester, but do so in the second and third trimester by 15% and 25%, respectively, in the pregnancies of women with normal protein synthesis and normal weight (23,24). Although it is not known how changes in protein synthesis during pregnancy affect maternal obesity, there is reduced protein anabolic response, consistent with insulin resistance, in obese women who are not pregnant. In a small scale study in which pregnant women with BMI of 21.0-29.0 kg/m2 were included, the mass of internal organs was found to positively correlate with the maternal protein cycle, contributing to greater length of the newborn baby (25).

The effective factor in the placental transport of protein is the sodium-dependent transporter family responsible for amino acid transport and expressed in the central nervous system. This factor, as expressed from placenta, affects fetal growth. The decrease in the activity of the system A transporter (SNAT1, SNAT2, SNAT4, also known as SCL38A1, SCL38A2, and SCL38A4) is associated with the restriction of fetal growth. Several past studies have demonstrated that the placental system A in pregnant women (BMI 30-39.9) reduced SNAT activity. This reduction of activity runs contrary to the hypothesis that suggests an increase in placental SNAT. The authors of the study are of the opinion that such a correlation can exist because the women included in this study were obese participants who had the gestational weight gain (GWG) recommended. The reduction in placental SNAT activity supports the hypothesis that infants born of obese mothers should have reduced body mass (26,27).

Obese women are at high risk of intraoperative and postoperative complications, which can be enumerated as postpartum hemorrhage, anesthesia complications, unsuccessful intubation, and retardation in the healing of postoperative injuries and infection, thromboembolism, and endomyometritis puerperium (28). Studies have shown that 25% of mortalities in pregnant women result from obesity. Regarding maternal mortalities, 18% are related with anesthesia and 80% with obesity (29,30). There are also studies investigating the effects of ethnicity on obesity and its side effects. In one study in African-American and Asian obese women, the rate of birth by cesarean section was found to be higher. The incidence of gestational diabetes in Latin and Asian obese women was reported to be twice as high as compared to other communities. In another study, Latin obese women, when compared with the women of other ethnic groups, were the only group with increased incidence of preeclampsia (31).

In a recent study, it was propounded that increased BMI is strongly correlated with low gestational weight, lower segment cesarean section, preeclamptic toxemia, pregnancy-induced hypertension and that, interestingly, this correlation is independent of maternal glucose levels. In addition, contrary to what is known, there are also studies which demonstrate absence of an important relationship between maternal BMI and losses of pregnancy (32). Sufficient evidence could not be found to indicate the relationship of GWG with neonatal glycemia, neonatal distress, hyperbilirubinemia, neonatal hospitalization, and other cases of infant morbidity. The evidence for the relationship between the major forms of pregnancy complications, such as GDM, and complications of hypertensive pregnancy and GWG is rather flimsy (33,34). In addition, the International Association of Diabetes and Pregnancy Study Group proposed in their study in 2012 that the blood sugar criteria for the diagnosis of GDM at the end of the oral glucose tolerance test in pregnant women should also be revised (35).

There is evidence to link increased maternal obesity with placental ultra-structural changes, accumulation of maternal macrophage and placental weight increase, vascular muscularity, and the expression of inflammatory cytokines. This placental response can help explain the short-term and long-term side effects observed in the children of obese mothers (36,37,38).

In the middle period of pregnancy (weeks 18-28), the relationship between GWG and adiposity in childhood was demonstrated. However, no such relationship was detected between childhood adiposity and GWG in women who had put on more than 500 gm a week after gestational week 28 (39).

In contrast to the findings of some studies, glucose is the main substance to cause obesity in the development of the fetus, a finding which confirms the view that maternal hyperglycemia throughout pregnancy is one of the most important factors which enable pregnancy complications in the obese population to be predicted. Children born to women with GDM are likely to develop macrosomia, shoulder dystocia, childhood obesity, and type 2 diabetes mellitus (DM) (40).

Stillbirth, fetal distress, congenital malformations (three-fold frequent), defects in the jugular vein and abdominal wall, intestinal defects, hydrocephaly, omphalocele, neural tube defect, macrosomia, shoulder dystocia, hypoglycemia, jaundice can be listed among the negative effects of maternal obesity in the fetal and neonatal period. These findings can be explained by the insufficiency in the distribution and absorption of the basic building substances (e.g. folic acid), by the elevation in the blood levels of triglycerides, uric acid, estrogen, and insulin, and hyperglycemia associated with insulin resistance caused by GWG. In a comprehensive retrospective study including obese and non-obese women with macrosomic newborns, obstetric complications in the obese group have been reported to be 3 times more frequent (17% vs. 6%) (41,42,43,44).

Obesity is associated with variations in cytokines and adipokines and with chronic inflammatory conditions. Adipose tissue, an active endocrine organ, is the source of pro-inflammatory cytokines such as adiponectin. Adiponectin is considered to be not only an important mediator which raises glucose sensitivity but also a substance which acts as a stimulant of glucose uptake by skeletal muscle (45,46). Adiponectin levels go down in healthy pregnant women. A low level of adiponectin, in turn, is associated with an increase in fetal growth. Adiponectin binds to receptor-2 in trophoblast cells, activating P38 MAPKPPAR-α, which is an insulin/insulin-like growth factor-1 (IGF-1) signal pathway inhibition. For this reason, it is not surprising that maternal serum adiponectin has an inverse relation with fetal growth (47). Oxide lipids can be cytotoxic and affect gene expression by activating cell nucleus. In addition, they can affect antenatal organ development and the response to environmental stimulants in the postnatal period. Moreover, exposure to a high level of lipid in intrauterine life can cause epigenetic changes in the metabolism genes and lipid sensitivity through molecular stimulation and transcriptional activation (48,49). Furthermore, obesity can increase the products of reactive oxygen resulting from increased oxidative stress in mitochondrial tissue. Oxidative stress and excess lipid combination result in increase of oxide lipids which, on the other hand, play role in some of the obesity-related side effects of pregnancy, such as oxide lipids trophoblast invasion and placental development, lipid metabolism, and preeclampsia (48,50).

Maternal obesity is also associated with apoptosis and decreased placental proliferation, increasing the tendency to develop side effects in the course of pregnancy. Experimental studies on animal models have demonstrated that a long-term diet rich in fat results in a change in placental vascularization, which, in turn, causes oxidative stress and increases the possibility of a hypoxic placenta. Such placental changes account for the increase in the number of stillbirths, the decrease in the number of surviving newborns, and for the increase in abnormal birth weights. Epidemiological studies have also detected a relationship between placental dysfunction and maternal metabolic syndrome (51,52,53). Lipid metabolism becomes impaired in adiposity. When hyperlipidemia arises, it reduces the prostacyclin level while increasing the thromboxane level. It is seriously considered that these changes affect the placenta causing fetal deaths (54). Serum leptin concentration is positively correlated with the storage of fat in the body. Maternal obesity is accompanied by placental leptin resistance and maternal hyperleptinemia which assist amino acid transfer and placental functions. IGF-1:IGFB3 ratio and leptin levels have been found to be higher in the cord blood of fetuses that are large in relation to their gestational age. High ratio of IGF-1:IGFB3 can also be one cause of abnormal fetal growth (26,55).

The incidence of dizygotic twins was found to be 1.1% in the offspring of mothers whose BMI was 30 and above, and 0.5% in those with a BMI below 25. However, no increase was detected in the incidence of monozygotic twins born to obese mothers (56). Maternal obesity disrupts iron transfer to the fetus owing to the increase in the level of hepcidin, in particular, and to the effect of a pro-inflammatory medium. Due to these changes, anemia often occurs in maternal obesity (57).

Maternal obesity increases the risk of early neonatal death and stillbirth. It has been demonstrated that an increase of 3 units or more in BMI during pregnancy considerably increases the risk of stillbirth and pregnancy-related complications including preeclampsia, gestational diabetes, gestational hypertension (21,58,59). In England, one-third of mothers with babies who are stillborn or die during the neonatal period are obese. According to the meta-analyses of 9 studies, stillbirths occur in obese women twice as frequently as they do in non-obese women (60). Again in England, a large-scale cohort study revealed that the incidence of stillbirths among obese women is 6.9/1000, while it is 4/1000 among those who are not overweight. The mechanism underlying this relationship is not clear. However, there are studies which indicate changes in lipid metabolism as a possible cause. Stillbirths may be related with hypertension or maternal obesity with gestational diabetes. Maternal obesity has been found to be related with the risk of mortality in the early neonatal period (Table 2) (21,58).

Significant conclusions have been drawn from studies on the relationship of perinatal mortality with ethnicity. In one such study in recent times, perinatal mortality rate in the offspring of black women proved to be twice as high as that of the offspring of white women. The risk of stillbirth was observed to increase by 30% in the class 1 obese group, and by 50% in the extremely obese group. In a retrospective study on the relationship between mortality in pregnancy with twins and maternal obesity, the researchers compared obesity in all twin pregnancies with women of normal weight and found that the number of stillbirths in the obese mothers was greater by 31%. When obese women pregnant with triplets were compared to their counterparts of normal weight, they had a 4 times greater risk of having stillbirths. These findings also demonstrate that the in utero survival rate in women pregnant with singleton or twins is higher than it is in pregnant women pregnant with triplets. This increase in risk suggests the existence of synergistic factors between fetal number and high BMI (61,62).

Although the correlation between obesity and stillbirth cannot be fully explained, some mechanisms regarding the issue have been proposed. It is possible to draw the conclusion that conditions such as apnea-hypoxia, retardation of fetal growth increasing the risk of fetal loss, and pregnancy-induced hypertension, which are all encountered more frequently in obese women than they are in women of normal weight, result in an increase in stillbirths. Impairment of the endocrine system and lipid metabolism causes increases in adiposity, which, in turn, results in hyperlipidemia in obese mothers. Hyperlipidemia increases the production of thromboxane while it reduces the secretion of prostacyclin. These changes lead to reductions in placental thrombosis and placental perfusion. In addition to insulin resistance, the decrease in fibrinolytic activity and perfusion increases the risk of thrombolysis considerably. The impairment of placental blood flow results in fetoplacental dysfunction. This phenomenon may reflect in clinic the increment of risk for stillbirth. Hypertensive diseases and DM are also among the factors which increase the risk of stillbirths (Table 2).

Cohort studies have revealed that, compared with women of average weight, obese women have more frequent elective preterm births; rate of preterm spontaneous births is also higher in the obese (63). This association changes with parity. In nulliparous women, the risk of requirement for elective preterm birth increases, but at the same time, the risk of spontaneous preterm birth decreases. The association of obesity with the risk of preterm birth is weak in multiparous women. However, the risk of preterm birth was shown to be increased in obese multiparous women (64).

Still, the relationship between maternal obesity and preterm birth is complicated, and other factors such as ethnicity, smoking, parity, and age-related factors are potentially involved in this relationship. Preeclampsia is the most important factor to increase the risk of elective birth in nulliparous women. In a large-scale cohort study in Scotland with 187,290 women, it was found that 40% of women who had morbid obesity and were subjected to elective preterm birth were preeclamptic (64). In the same study, it was also found that among multiparous women, the risk of preterm birth increases as BMI increases, while the risk of spontaneous preterm birth decreases, and preterm birth is only a weak correlate of preterm birth in multiparous obese women. How obesity reduces the risk of spontaneous preterm action while all the risks causing prematurity increase in a nulliparous morbidly obese pregnant woman is not fully understood. The mechanisms of obesity in reducing the risk of spontaneous preterm births cannot be explained adequately. It may be speculated that it is related with the reduction in the level of spontaneous uterine activity in obese women compared with normal or thin women (65,66).

It is possible to further analyze the pathogenesis of spontaneous preterm action. In the Scottish cohort study, it was found that the risk of spontaneous preterm action is low in obese women when membranes are intact, but, with the preterm rupture of amniotic membranes, the risk of preterm birth increases. It is thought that this condition may be related with metabolic syndrome (endothelial dysfunction, systematic inflammation, and insulin resistance) associated with acute chorioamnionitis, light or subclinical genitourinary system infection, or obesity. Again in the same study, from the analysis of more than 3000 preterm infants, it was understood that spontaneous preterm births, as a result of the rupture of amniotic membranes, are rather frequent in obese women, and that the risk of spontaneous preterm labor pains, with intact membrane, is reduced in obese women. The mechanism presumed to exist for these relationships has not yet been explained, but the presumed possible mechanisms may be obesity-related metabolic syndromes, subclinical infections of low grade, or acute chorioamnionitis (67).

Preeclampsia, which occurs in obese pregnant women with a greater incidence than in controls, is the most important problem leading to risky births in nulliparous women (Table 3). Obesity increases the birth risk triggered by preeclampsia and hypertension. An increase of 5-7 kg/m2 in BMI gives rise to twice as great an increase in the rate of preeclampsia (68,69).

Among the congenital structural anomalies resulting from maternal obesity, neural tube defect and congenital heart disease are of particular importance (Table 4) (65). In a comprehensive study in 2011, the rate of children born with congenital malformation to obese mothers was found to be 2% compared to 0.8% for children born to mothers with normal BMI. Talipes equinovarus and facial defects are also among the main congenital malformations encountered in the offspring of obese women. These differences in rate of congenital malformations have been reported to be unrelated with maternal glucose levels. In addition, no relationships were found between maternal BMI or glucose levels and neonatal hypoglycemia, jaundice, respiratory distress, admission to neonatal intensive-care unit, and finally, fetal death (32). In a meta-analysis published in 2009, children born to obese mothers were compared with those born to mothers with normal BMI (Table 4) (65).

The risk of neural tube defects starts to increase with weight gain before pregnancy, and possibly folate supplementation does not diminish this increase. Observational data have indicated that the risk of neural defects resulting from obesity is independent of the effect of standard daily folate supplement (>400 mcg) (70,71). The consensus reached in United Kingdom suggests that women with a BMI above 30 kg/m2 and planning to become pregnant receive 5 mg of folic acid daily, starting at least one month before conception and continuing throughout the first trimester. The sensitivity of ultrasonography (USG) decreases in overweight and obese women owing to technical difficulties, which, in turn, detracts from its accuracy. While the risk of congenital anomaly after normal USG is 1/250 in women with BMI below 25, it is 1/100 in obese women (72).

Interestingly, gastroschisis occurs less frequently in children born to obese mothers. However, this is thought to be related with maternal age. The incidence of obesity rises with age and low maternal age is a risk factor in gastroschisis (73). Various mechanisms are thought to contribute to the relationship between structural anomalies and maternal obesity.

While the relationship of congenital anomalies with maternal obesity may be partly due to the fact that some obese women have DM, it was found that the relationship between maternal obesity and neural tube defects is similar in systematic studies which specifically exclude women with DM or provide subgroup data for women without DM (73).

Even when such pregnancy complications as preeclampsia and diabetes are excluded, the intrapartum risk of morbid prognosis in obese women and in their babies is high compared to those who are not overweight (Table 5) (74).

Obese women are at high risk for intrapartum complications. Their need for surgical birth and support is high. Among the reasons for this is the delay in the development of labor pain, the birth being rendered difficult by fetal macrosomia, intrauterine growth retardation, or anus presentation. A wide variety of factors, which include macrosomia, can contribute to these adverse results. Among these factors are inadequate contractions of the uterus and the failure of labor to progress owing to macrosomia (16).

The frequency of fetal macrosomia, independent of diabetes existing previously but arising during pregnancy, is almost two-fold in obese pregnant women compared to those who are not overweight. Dystocia, birth trauma (fractures and nerve paralysis), perinatal asphyxia, and hospitalization occur more frequently in large sized neonates. On the other hand, in one comprehensive case-control study from Canada, which included 45,877 vaginally born babies exclusively, no relationship was found between maternal obesity and fetal weight, which was estimated to be within the normal ranges, nor between maternal obesity and dystocia (75).

Metabolic complications such as hypoglycemia, glucose dysregulation, as well as disorders in insulin, lipid, and aminoacid metabolism play an important role in development of pregnancy complications and increased maternal adiposity, and also of undesired perinatal side effects. The transplacental passage of glucose is significantly correlated with glucose concentration in maternal blood. Therefore, even a slight change in the glucose in the maternal circulation can be transferred to the fetus (76).

The diurnal and nocturnal glucose profiles of obese pregnant women with normal glucose tolerance are higher than those of their counterparts with normal body weight, by virtue of harmony of the potential mechanisms mentioned above, a finding which, in turn, makes the exposure of the fetus to hyperglycemia greater (77).

Obese women have higher insulin resistance than women with normal body weight (22). Apart from this, there is a linear relationship between maternal BMI and the other parameters related to obesity including glucose intolerance. The same relationship exists among elevated cord C-peptide levels and glucose intolerance and increased birth weight.

Kalk et al (78) monitored 505 infants of non-diabetic obese mothers and found that hypoglycemia occurs in 26% of these infants, while it was noted to occur in 9% of infants of mothers who were mildly overweight or of normal body weight and in 6% of babies of thin mothers. These findings suggest that babies born to obese mothers should also be monitored closely for hypoglycemia, as in the case of children born to diabetic mothers.

Vitamin D uptake mechanisms, through the interaction of megaline and eubilin which possess binding protein and receptors, are thought to be immature in the newborn (79). The placental passage of 25-hydroxy (OH) vitamin D in obese women is decreased. Therefore, even if mothers have the same level of 25-OH vitamin D, the levels in the cord blood of their offspring were reported to be lower than those of the offspring of mothers with normal weight. Other researchers have also observed that the levels of cord blood 25-OH vitamin D closely related to maternal 25-OH vitamin D, maternal obesity, maternal age, and neonatal obesity. A correlation has also been detected between maternal obesity, 25-OH vitamin D nutritional status, and adiposity in the neonatal period which is capable of affecting 25-OH vitamin D activity in childhood and adulthood (80).

In various studies, a positive association has been found among the degree of obesity in childhood and adolescence and weight gain during pregnancy. It has also been demonstrated that this adiposity, being increased at birth, is a risk factor for metabolic dysregulation and for childhood obesity (39,81,82,83,84). Obesity is a significant factor for the development of insulin resistance and metabolic syndrome in the adult. In fact, β-cell dysfunction and insulin resistance occur more frequently in obese women than in non-obese women (85). These findings show that obese women with genetic disposition are at higher risk of developing type 2 DM.

In utero insulin resistance was first suggested in 2009 in a study on the fetuses of obese women, which included measurements of glucose and insulin concentration in the umbilical cord. The findings corroborate the concept of fetal programming and its possible impact in subsequent periods of life (86). The first child of a woman with high BMI during pregnancy was reported to have a higher proportion of fat than the younger siblings, demonstrating a relationship between adiposity and maternal parity. The cause of the high mass of fat, which occurs more frequently in the first birth, is, in all probability, due to the re- regulation of the leptin and glucocorticoid axis in the adipocyte, which contribute to the increase in adipogenesis throughout the gestational period (87,88).

In another comprehensive longitudinal cohort study with infant participants, the obesity prevalence of 16-year-old children born to obese mothers and mothers with GDM was found as 40% and 26%, respectively. This prevalence has been demonstrated to persist among not only the children of mothers with GDM, but also those of obese mothers. In animal studies, hypothalamic neuronal changes were observed in the young of diabetic obese rats, caused by the intrauterine medium, which increases the risk of diabetogenic state and obesity. These data have not only provided the justification for the hypothesis of an in utero programming of metabolic syndrome in obese women’s children but have also provided clues to the researchers on the risk of prenatal disease (89).

At birth, infants born to overweight or obese mothers have a greater amount of adipose tissue than those born to mothers of normal weight. However, there are also data indicating that infants born to overweight or obese mothers are normal in the first 15 days, but fall behind normal children in terms of gaining weight, increase in height, and increase in adipose tissue in the first 3 months. Studies have shown that a programmed tendency to childhood obesity, leading to increase in the risks for childhood obesity, type 2 DM, and metabolic syndrome in adult life, may be related with low socio-economic level which prevents access to healthy food, especially when combined with the paucity of postnatal breast feeding (21,90,91,92,93).

GDM is a predisposing factor in the development of obesity and being overweight throughout the childhood period. The probability that macrosomic infants, in particular, will be obese in their future life is rather high. From the community health perspective, it is crucial that these children should be monitored closely for possible metabolic and cardiovascular risk, and that the necessary precautions should be taken (21,90). Transcriptional regulation in childhood and programming in the early period of life contribute to retardation in development, deficits in linguistic skills, and other neurological disorders. In recent years, it has been demonstrated that the probability of obese mothers having autistic children has increased 1.7-fold, and the probability of their having children with developmental retardation has increased 2-fold (94,95).

It has been proposed that high maternal BMI, notably early in pregnancy, is a predisposing factor in the development of schizophrenia in children, probably due to the high levels of pro-inflammatory cytokines during the second trimester (96,97). A large number of studies on animal and human subjects have demonstrated that maternal obesity during pregnancy is related with postnatal lifelong programming of children for chronic diseases, including aging, neurodevelopmental retardation, cancer, osteoporosis, type 2 DM, metabolic syndrome, and cardiovascular diseases. In a study in which telomere lengths were measured in 1122 women aged 18-76 years, obesity was found to aggravate the aging process through telomere erosion.

Obesity and excess weight gain before pregnancy raises the rate of miscarriages, as well as the rates of obstetrical and neonatal complications. These complications, in turn, result in a lower quality of health. It has been demonstrated that, in addition to adverse consequences for the mother, exposure to maternal obesity in the intrauterine period is an important risk factor for development of chronic diseases such as cardiovascular diseases, metabolic syndrome, and type 2 DM in childhood and adolescence.

Fetal programming of metabolic functions by means of physiological and epigenetic mechanisms causes interactions between generations, and obesity can acquire permanence in posterity. Therefore, it is of vital importance that ideal weight gain be achieved, care be taken for the prevention and/or right management of obesity if this vicious circle is to be broken, and the short- and long-term serious negative effects on the fetus and mother are to be avoided.

In conclusion, it can be stated that reducing the adverse effects of maternal obesity is a community health problem. More studies are required to assess the effects and reliability of weight management programs in women in the reproductive age group. The need for future studies on the negative side effects of maternal obesity and overweight on fetal growth and development, as well as for studies assessing the approaches which restrict weight gain in pregnancy and the postpartum period is of vital importance.

**Ethics**

Peer-review: Internal peer-reviewed.

## AUTHORSHIP CONTRIBUTIONS

Concept: Levent Korkmaz, Osman Baştuğ, Selim Kurtoğlu, Design: Levent Korkmaz, Osman Baştuğ, Selim Kurtoğlu, Data Collection or Processing: Levent Korkmaz, Selim Kurtoğlu, Analysis or Interpretation: Levent Korkmaz, Osman Baştuğ, Selim Kurtoğlu, Literature Search: Levent Korkmaz, Selim Kurtoğlu, Writing: Levent Korkmaz.

Financial Disclosure: The authors declared that this study received no financial support.

## Figures and Tables

**Table 1:**
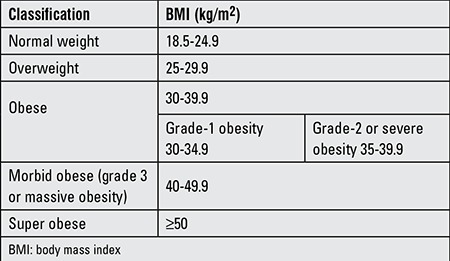
Classification of obesity based body mass index (2)

**Table 2:**
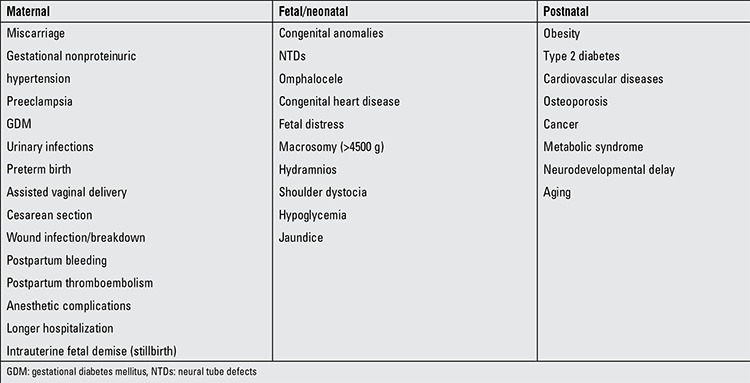
Short- and long-term complications associated with maternal obesity (21)

**Table 3:**
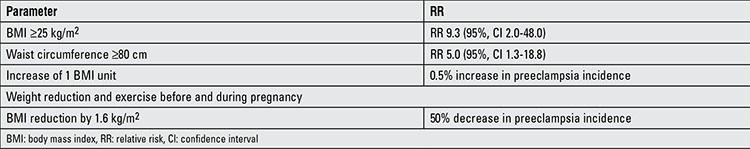
Maternal obesity and relative risk of developing preeclampsia (68,69)

**Table 4:**
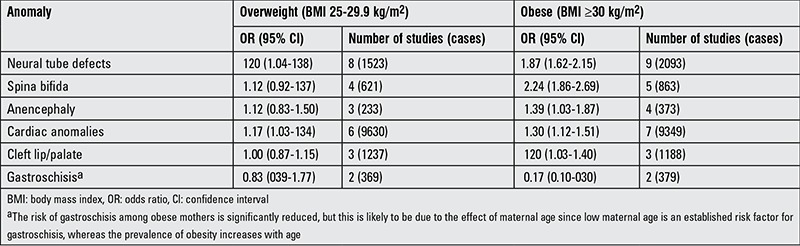
Relationships between specific congenital anomalies associated with maternal obesity and overweight state (vs. women with normal body mass index) (65)

**Table 5:**
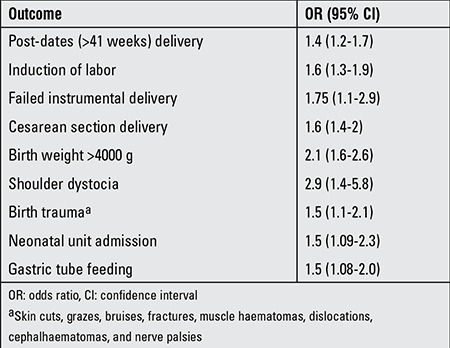
Risk of more common adverse perinatal outcomes in obese women vs. women with normal body mass index (primigravid singleton otherwise uncomplicated pregnancies) (74)
